# SARS-CoV-2 Viral Load Is Correlated With the Disease Severity and Mortality in Patients With Cancer

**DOI:** 10.3389/fonc.2021.715794

**Published:** 2021-08-18

**Authors:** Maha Al-Mozaini, Abu Shadat M. Noman, Jawaher Alotaibi, Mohammed Rezaul Karim, A. S. M. Zahed, A. T. M. Rezaul Karim, Khaldoun Alromiah, Syed S. Islam

**Affiliations:** ^1^Department of Infection and Immunity, King Faisal Specialist Hospital and Research Centre, Riyadh, Saudi Arabia; ^2^Department of Biochemistry and Molecular Biology, University of Chittagong, Chittagong, Bangladesh; ^3^Department of Medicine, King Faisal Specialist Hospital and Research Centre, Riyadh, Saudi Arabia; ^4^Department of Medicine, Park View Hospital, Chittagong, Bangladesh; ^5^Clinical Genomic Centre, King Faisal Specialist Hospital and Research Centre, Riyadh, Saudi Arabia; ^6^Department of Molecular Oncology, King Faisal Specialist Hospital and Research Centre, Riyadh, Saudi Arabia; ^7^School of Medicine, Al-Faisal University, Riyadh, Saudi Arabia

**Keywords:** cancer, chemotherapy, SARS-CoV-2, viral load, mechanical ventilation, intensive care unit, incubation period, serial interval

## Abstract

The correlation between severe acute respiratory syndrome coronavirus-2 (SARS-CoV-2) viral load and risk of disease severity in cancer patients is poorly understood. Given the fact that cancer patients are at increased risk of severe coronavirus disease 2019 (COVID-19), analysis of viral load and disease outcome in COVID-19-infected cancer patients is needed. Here, we measured the SARS-CoV-2 viral load using qPCR cycle threshold (Ct) values collected from 120 noncancer and 64 cancer patients’ nasopharyngeal swab samples who are admitted to hospitals. Our results showed that the in-hospital mortality for high viral load cancer patients was 41.38%, 23.81% for medium viral load and 14.29% for low viral load patients (*p* < −0.01). On the other hand, the mortality rate for noncancer patients was lower: 22.22% among patients with high viral load, 5.13% among patients with medium viral load, and 1.85% among patients with low viral load (*p* < 0.05). In addition, patients with lung and hematologic cancer showed higher possibilities of severe events in proportion to high viral load. Higher attributable mortality and severity were directly proportional to high viral load particularly in patients who are receiving anticancer treatment. Importantly, we found that the incubation period and serial interval time is shorter in cancer patients compared with noncancer cases. Our report suggests that high SARS-CoV-2 viral loads may play a significant role in the overall mortality and severity of COVID-19-positive cancer patients, and this warrants further study to explore the disease pathogenesis and their use as prognostic tools.

**Graphical Abstract:**
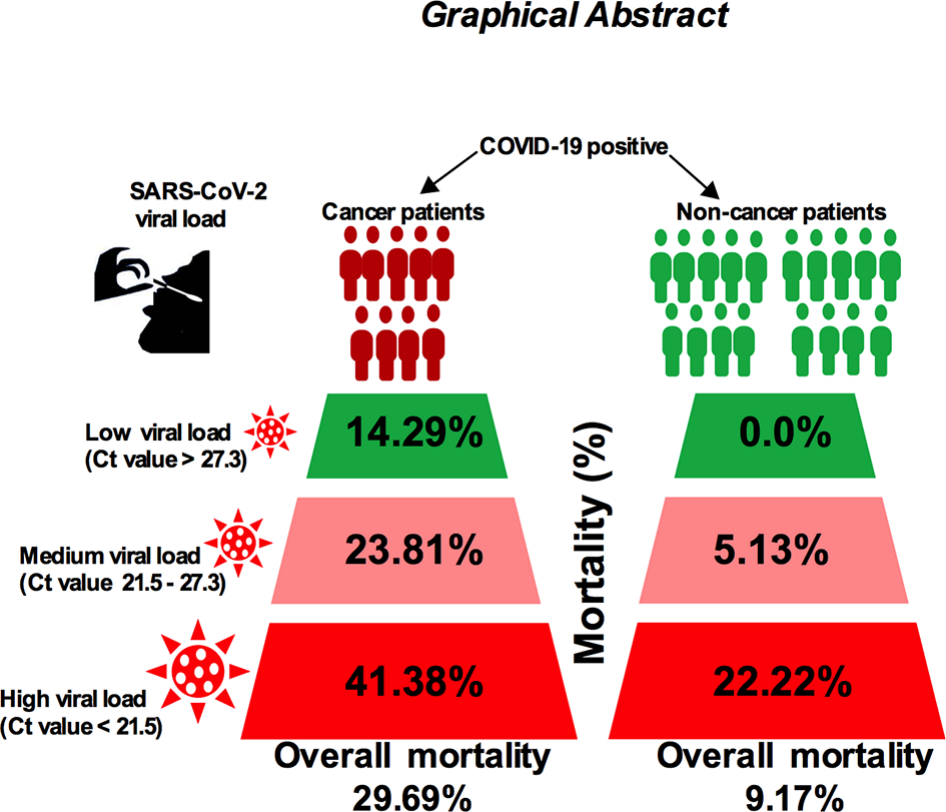


## Introduction

Individuals who are infected with severe acute respiratory syndrome coronavirus 2 [SARS-CoV-2; coronavirus disease 2019 (COVID-19)] demonstrate a heterogeneous presentation of symptoms and severity depending on the history of active malignancies. The COVID-19 symptoms range from mild to severe and may have deadly outcomes. Individuals with a history of cancer are more susceptible to COVID-19 infection and have been identified as having an increased risk of mortality and morbidity due to overall poor health status and systemic immunosuppressive conditions (1, 2).

Recent reports suggest that age, male sex, smoking status, and comorbidities (hypertension, cardiovascular disease, and diabetes) have been identified as risk factors that have a severe impact on patient mortality in patients with cancer (3, 4). Among other comorbid factors, cancer was identified as a susceptible group and have been identified as having an increased risk of infection with COVID-19 with increased risk of death and severe outcomes. These severe phenotypes require special attention with higher intensive care, correlating with rapidly deteriorating patient conditions and increased risk of death (5–7) with a clear contrast between the patients with and without cancer. Furthermore, the clinical phenotypes of patients with cancer and the effects of anticancer treatment greatly influence the outcome of the patient’s severity and survival (2, 8).

It was reported that high SARS-CoV-2 viral load was independently associated with in-hospital mortality among COVID-19-positive population (7–9). In addition, high SARS-CoV-2 viral load in patients with cancer have been reported recently (8, 10). Although this report highlighted the relationship between viral loads and mortality of cancer and noncancer patients, the impact of viral loads on outcomes of patients with cancer with a specific cancer type and anticancer treatment require additional study to determine whether SARS-CoV-2 viral load may precisely predict the outcome of cancer patients particularly in patients who are undergoing chemo and/or radiotherapy.

We aimed to illustrate the clinical characteristics and outcome of patients with and without cancer and presented evidence of the effects of SARS-CoV-2 viral loads among patients with and without cancer. Here, we show that SARS-CoV-2 high viral loads adversely affect the certain type of cancer patients particularly those who are under chemotherapeutic treatment which may provide a predictive tool for cancer patient mortality.

## Methods

### Study Design and Patients

In total, 64 patients with cancer confirmed with COVID-19 who were admitted to King Faisal Specialist Hospital and Research Centre, Riyadh, Saudi Arabia and Park View Hospital, Chittagong, Bangladesh between June 30, 2020 and August 7, 2020 were included in this study. A total of 120 COVID-19-positive noncancer adult patients admitted by the same hospitals and same time period were included in this study as a control group. We excluded patients who displayed radiological or clinical diagnosis of COVID-19 but without a positive RT-PCR result. For the cancer cohort, patients with active cancer were defined as those undergoing anticancer treatment with curative, radical, adjuvant, or neoadjuvant therapy or treated in the last 12 months with radiotherapy, surgery, and chemotherapy. The dataset contained mainly information on exposure time and time of symptoms onset, and as such all cancer and noncancer cases by definition are symptomatic. Four clinical outcomes were monitored up to August 30, 2020. We have classified disease severity in four main categories: (a) “Mild cases” if the patients did not show any serious symptoms described in moderate, severe, and deceased categories but positive nasopharyngeal swab RT-PCR test and did not require serious medical intervention; (b) “moderate cases” if the patients showed fever, some respiratory symptoms, and any evidence of pneumonia by radiography; (c) “severe cases” defined as cases experiencing any of the following symptoms: breathing rate of 30 or more breaths/min, the oxygen saturation level below 93%, one or multiple organ failures, requiring intensive care unit (ICU) and invasive mechanical ventilation support (IVS), a ratio of the partial pressure of arterial oxygen to the fraction of inspired oxygen (PaO_2_:FiO_2_) of less than 300 mmHg, or infiltrates in more than 50% of the lung field within 24 to 48 h; and (d) “deceased cases”, i.e., patients admitted to the hospitals with COVID-19-related symptoms who died during their hospital stay. This study was approved by the central ethics committee of the Bangladesh Medical Research Council/Park View Hospital [study # 2021-2023/62(1-20)] and King Faisal Specialist Hospital and Research Centre (RAC # 2200031)—a waiver of informed consent from patients was also approved. All research was performed in accordance with the relevant ethical guidelines and regulations.

### SARS-CoV-2 Viral RNA Load Measurements

Nasopharyngeal swab samples collected from June 30 to August 7, 2020, from cancer and noncancer patients and samples were stored in room temperature and processed within a reasonable time of collection. Nucleic acid was extracted from samples using MOLgen-SARS-CoV-2 and Sansure Biotech SARS-CoV-2 assay protocols according to the manufacturer’s instructions. Extracted RNA was eluted using elusion buffer. Levels of SARS-CoV-2 viral load were determined using US CDC real-time RT-PCR primer and a probe set for 2019-nCoV N1. Ct values obtained from the N1 primer set were converted to RNA copies/ml using N1 quantitative PCR (qPCR) in 10-fold dilution standard curve as described previously (11, 12). Values were log_10_ transformed. We converted Ct values into quantitative assessments of viral load—high, Ct value <21; medium, Ct value 21–26; and low, Ct value >26. These cutoff values (high, medium and low) were used to determine the risk of severity and death of all patients used in this study.

### Data Collection and Procedures

A standardized case-report form was used to collect the patient’s clinical data. Patients cancer characteristics, detail treatment history was retrieved from the medical records books. We collected data from patients by our data collection team with the assistance of participating hospitals registry personnel. Patient demographics, cancer details, cancer treatment information, age, sex, number of comorbidities which require active treatment, surgical history and diagnosis of COVID-19, cancer status, and treatment during hospitalization were collected.

### Outcomes

We considered and analyzed four clinical outcome/endpoints: severe illness requiring admission to the hospital, death, admission to ICU, and needing invasive mechanical ventilation support (IVS).

### Incubation Period Analysis

We recorded daily mortality and hospitalization incidence and plotted the cumulative numbers of confirmed and discharged cases based on symptom onset date for noncancer and cancer cohorts. We excluded cases that did not have a definitive symptom onset date. We generated a source of disease plot based on the best information available during the study period. For incubation period analysis, we used direct estimation methods (13) based on the earliest and latest possible exposure times and reported symptom onset times. The parametric distributions (gamma distribution, Weibull distribution, and lognormal distribution) of incubation period was estimated using interval censoring using maximum likelihood of a time falling in a defined window. We used interval censoring because it is not possible to know the exact time of exposure.

### Serial Interval Analysis

We estimated serial intervals in a direct method as the difference between the symptom onset dates. We excluded asymptomatic cases as well as those cases that do not have exact symptom onset dates. We fitted the normal, log normal, gamma, and Weibull distributions using R packages “fitdstrplus” by maximum likelihood of a time falling in a defined window. We computed each fit distribution using Akaike information criteria (AIC) scores and calculated confidence intervals for parameters from 1,000 bootstrap replicates (14).

### Statistical Analysis and Data Visualization

All statistical analyses were performed using R (version 4.0.3; October 10, 2020) statistical software and “ggplot2” package for generating graphs. We assessed and reported clinical outcomes of COVID-19-positive noncancer and cancer patients whether patients died or discharged and the effects of anticancer therapy on the underlying conditions of patients. We calculated the percentages of patients in each category for the categorical data. The Wilcoxon rank sum test was used for continuous data, and two-sided Fisher’s exact test was used to compare categorical data for different categories of data without multitest. Multivariate logistic regression analysis was used to estimate the odds ratio (OR) and 95% confidence interval (CI) of each factor of interest with confounders/outcomes. The OR was adjusted to chronic renal disease, cardiac disease, diabetes, hypertension, asthma, and pulmonary disease. A two-sided *p-*value <0.05 was used to indicate the statistical significance. We constructed a multivariate logistic analysis model to identify variables that were independently associated with high and low viral load. Correlation analysis was performed using Spearman’s rank-based testing.

## Results

### Clinical Characteristics of Patients Among Noncancer and Cancer Patients

**Figure 1:**
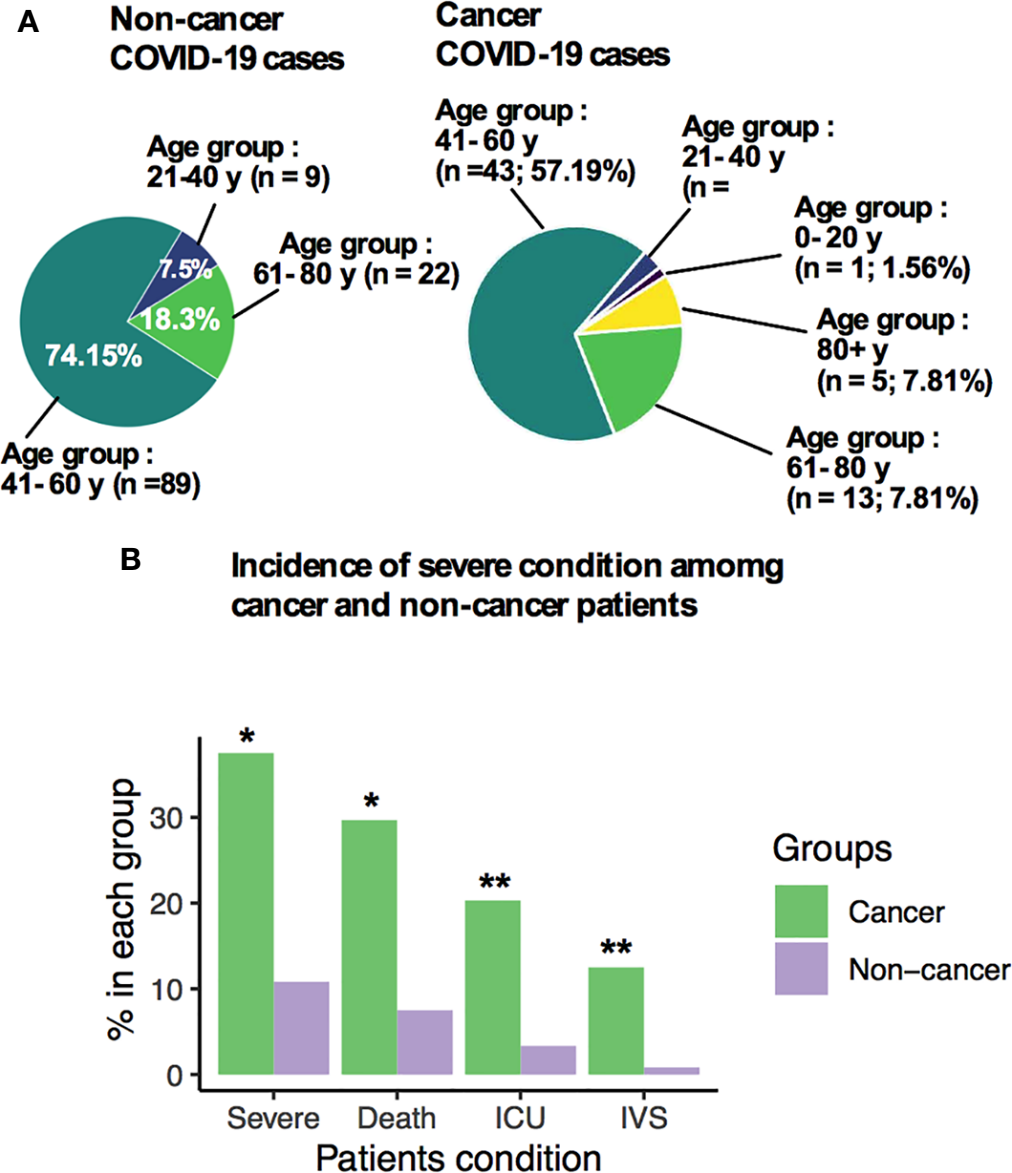
Clinical characteristics of patients among non-cancer and cancer patients. **(A)** Age distribution of patients with non-cancer and cancer patients. **(B)** Incidence of severe condition among non-cancer and cancer patients. *P ≤ 0.05; **P ≤ 0.01.

We have obtained and analyzed 64 RT-PCR-confirmed COVID-19-positive cancer patients from June 30, 2020 to August 7, 2020 from Park View Hospital (*n* = 48), Bangladesh and King Faisal Specialist Hospital and Research Centre (KFSHRC; *n* = 16), Riyadh, Saudi Arabia. In addition, we have collected information of 120 COVID-19-positive patients without the history of cancer from the same hospitals (KFSHRC *n* = 60 and *n* = 60 Park View Hospital). The latter group was used as a control group to compare the parameters between cancer and noncancer patients. The median age of cancer patients was 55 [interquartile range (IQR) 10.5] and 53 (IQR 9.0) for noncancer patients (*p* < 0.0001; **Table 1**). In our patient population, the range of age distribution for cancer and noncancer was close. **Figure 1A** shows the age distribution of patients with noncancer and cancer. In total, the two cohorts comprised 123 male and 61 female cases, and male to female ratio is 2:1 (**Table 1**). When assessing the patient’s comorbid factors, diabetes ranks the top [31/120 (25.83%) *vs*. 13/64 (20.31%)] for noncancer cases. However, hypertensive condition was deadliest in cancer cases [19/64 (29.69%) *vs*. 21/120 (17.5%)] than noncancer cases. Typical COVID-19-related signs and symptoms, such as, fever [65/120 (54.17%) *vs*. 29/64 (45.31%)] and cough [43/120 (35.83%) *vs*. (26/64 40.63%)] were the highest in both cancer and noncancer cases. **Table 1** summarizes the clinical characteristics of patients.

**Table 1:** Characteristics of COVID-19-positive noncancer and cancer patients.

Characteristics	COVID-19 patients without cancer (n = 120)	COVID-19 patient with cancer (n = 64)	p-Values
Age (years, median/IQR)	53.0/9.0	55/10.5	<0.0001
***Sex***			
Male	75/62.5%	48/75.0%	0.63
Female	45/37.5%	16/25%	0.59
***Source of infection***			
Hospital	41/34.17%	29/45.31%	0.16
Family	14/11.67%	15/23.44%	0.77
Unknown	65/54.17%	20/31.25%	0.15
***Underlying medical conditions***			
Chronic renal disease	9/7.5%	¾.69%	0.69
Cardiac disease	11/9.17%	15/23.44%	0.003
Diabetes	31/25.83%	13/20.31%	1.0
Hypertension	21/17.5%	19/29.69%	0.05
Asthma	5/4.17%	1/1.56%	1.0
Pulmonary disease	4/3.33%	1/1.56%	1.0
No known disorders	39/32.5%	12/18.75%	0.07
***Hospitalization status***			
Discharged	109/90.33%	45/70.31%	0.56
Deceased	9/7.5%	19/29.69%	0.04
***Duration in hospital stay (days)***	13.6	23.8	0.0001
***Typical COVID-19 signs and symptoms***			
Fever	65/54.17%	29/45.31%	0.03
Cough	43/35.83%	26/40.63%	0.02
Nausea	3/2.5%	6/9.34%	0.07
Fatigue	2/1.67%	3/4.69%	0.08
Sore throat	7/5.83%	2/3.13%	1.0
***Treatments during hospitalization***			
Antiviral treatment	49/40.83%	19/29.61%	0.39
Oxygen therapy	19/15.83%	23/35.94%	0.0001
Antibiotic treatment	46/38.33%	25/39.06%	0.04
Invasive mechanical ventilation	6/5.0%	13/20.31%	0.04
***Cancer type***			
Breast cancer	N/A	16/25.0%	N/A
Ovary	N/A	5/7.81%	N/A
Lung/NSCLC	N/A	9/14.06%	N/A
Hematologic cancer	N/A	3/4.69%	N/A
Bladder	N/A	6/9.38%	N/A
Colon	N/A	7/10.94%	N/A
HNSCC	N/A	6/9.38%	N/A
Rectal	N/A	5/7.81%	N/A
Pancreas	N/A	3/4.69%	N/A
Esophageal	N/A	4/6.25%	N/A

N/A, Not applicable.

Overall, intensive care unit (ICU) admission, disease severity, and invasive ventilation support requirements was urgently needed for cancer patients compared with noncancer patients (**Figure 1B**), and death was principally attributed to COVID-19-positive cancer patients. In the cancer cohort, the death rate was strikingly higher (29.69%) compared with noncancer cases (7.5%; **Figure 1B**, *p* < 0.05) given the fact of similar age distribution between cancer and noncancer patients. The requirement of ICU admission was greater (20.31%, *p* = 0.01) in cancer patients than the noncancer cohort, showing a higher rate of having a severe condition (37.5%; *p* = 0.05), and needing and utilizing invasive ventilation support (IVS; 12.5%; *p* = 0.01; **Figure 1B**).

As of August 15, 2020, 28 (10.32%) patients had died in the noncancer (9/120; 7.5%) and cancer (19/64; 29.69%) cohorts, all within 15–18 days with reports stating that the death was substantially attributed to COVID-19. The patients who died from COVID-19 infection in the cancer and noncancer patients were mostly older age group. The age distribution of death rate in cancer patients was between 40 and 59 years, while for noncancer patients, it was between 40 and 69 years (**Supplementary Figure S1A**). We also stratified the severe condition of all cancer and noncancer patients by age group. In general, the requirement of ICU support for cancer patient was higher in the older age group (40–69 years), but in the noncancer group, only patients aged between 60 and 79 years needed ICU support (**Supplementary Figure S1B**). The symptoms of severity in cancer and noncancer patients increased with age (**Supplementary Figure S1C**), and ventilation support was proportionally higher in cancer patient age groups between 40 and 69 years (**Supplementary Figure S1D**). In contrast, no invasive ventilation support was needed for noncancer patients at any given age group (**Supplementary Figure S1D**).

The overall pooled OR of all identified comorbidities for noncancer patient was 0.45 (95% CI: 0.240.86, **Supplementary Figure S1E**). A forest plot of the potential underlying conditions is shown in **Supplementary Figure S1E**. COVID-19-positive noncancer patients with cardiac disease (odd ratio: 0.33, 95% CI: 0.09–1.27), diabetes (odd ratio: 0.62, 95% CI 0.16–2.43), and hypertension (odd ratio: 0.82, 95% CI: 0.28–2.42) remained the deadliest comorbidity factors among all noncancer patients (**Supplementary Figure S1E**). According to multivariate regression analysis, COVID-19-positive cancer patients had overall higher OR (OR: 2.59; 95% CI: 1.47–5.26; *p* < 0.001; **Supplementary Table S1A**) for death, ICU admission (OR: 4.22; 95% CI: 3.17–6.26, *p* < 0.001; **Supplementary Table S1B**) and severe symptoms (OR: 2.26; 95% CI: 1.77–4.61, *p* < 0.01; **Supplementary Table S1C**) when considering with other underlying conditions.

### Incubation and Serial Interval Period Analysis for Noncancer and Cancer Patients

**Figure 2:**
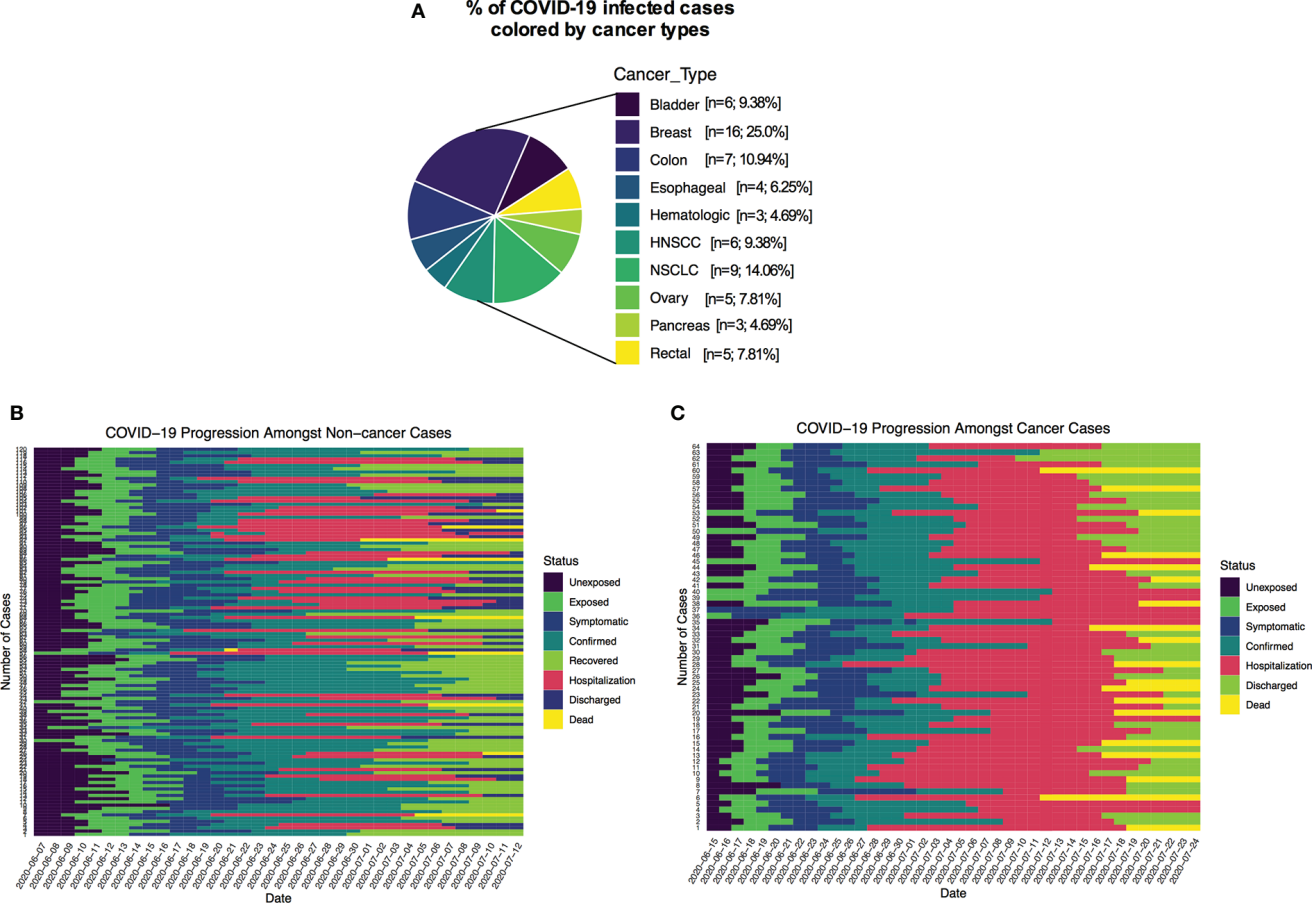
**(A)** Percentage of COVID-19 positive cancer cases colored by cancer types. **(B, C)** Disease timelines: date at which cases is unexposed, exposed, symptomatic, date of confirmed, recovered, date of hospitalization, discharged and deaths of cancer and non-cancer COVID-19 cases.

Types of malignancies in the COVID-19 patients may greatly influence the patient’s outcomes. To expand our understanding of patient malignancies and symptom onset between cancer and noncancer patients, we have investigated the incubation period and serial interval time. Our study composed of several cancer types where breast cancer was overrepresented (16/64, 25.0%), followed by lung (9/64, 14.06%), colon (7/64, 10.94%), and head and neck cancer (6/64, 9.38%) (**Figure 2A**). The overall death rate in cancer patients was 29.69%. The death rate among lung cancer patients was higher (7/64, 10.93%), while hematologic and ovarian cancer patients had the second highest death rate (3/64, 4.69%; 3/64, 4.69%). Breast, colon, and gastrointestinal cancer each had two deaths out of 64 (2.94%) (**Supplementary Table S2**). The patients who had a high death rate also were accompanied with severe and critical symptoms, longer ICU supports, and invasive mechanical ventilation requirements (**Supplementary Table S2**).

**Figure 3:**
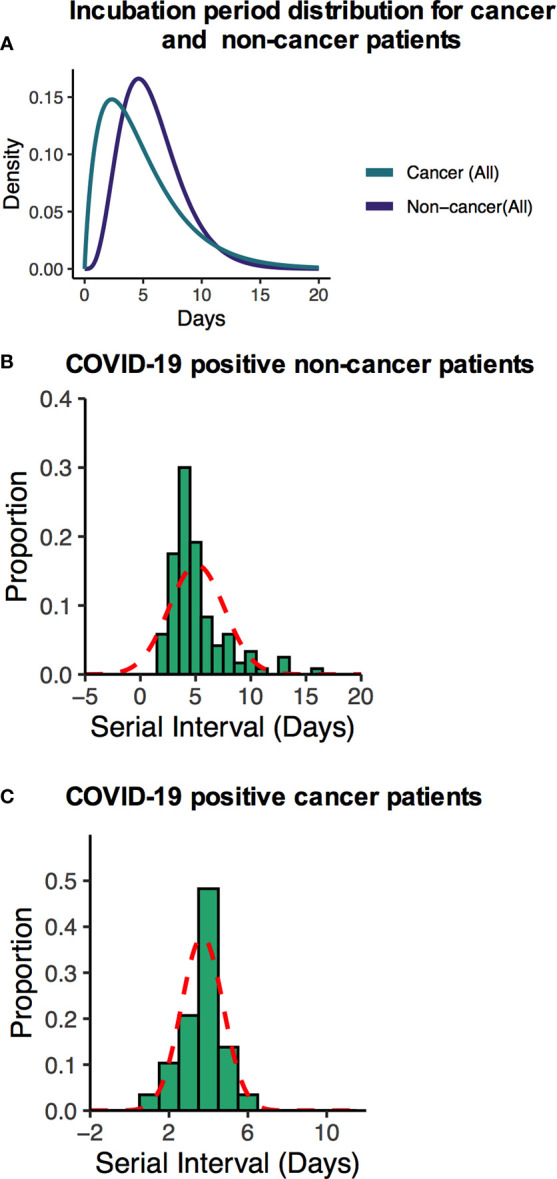
**(A)** Fitted gamma COVID-19 incubation period distributions for all non-cancer and cancer cases including cancer specific cases. **(B, C)** Serial interval distribution of COVID-19 infection cases among cancer and non-cancer cases.

Many recently published manuscripts reported that the mean incubation period for COVID-19-positive cases was between 5.6 and 6.7 days and the pooled parameter estimates the median period of 5.1 and 11.7 days (15). In the noncancer cohort, we find that the median incubation period is 5.41 days with the gamma distribution: shape 4.84 (95% CI: 3.41–5.93) and scale 1.2 (95% CI: 0.81–1.34). On the other hand, the mean incubation period is 5.81 (95% CI: 5.17–6.50). In the cancer cohort, the median incubation period is 4.10 days with a gamma distribution shape of 1.89 (95% CI: 1.32–2.42) and scale 2.61 (95% CI: 1.47–3.79). The calculated mean is 4.99 days (95% CI: 3.60–6.71) days. Summary of the results is presented in **Supplementary Table S3** and fitted Weibull and log normal distributions is in **Supplementary Table S4**. Although our results are consistent with recently published data for the noncancer group, the cancer patient’s incubation time was shorter than their noncancer counterparts. These results suggest that the estimated mean incubation period for cancer patients is likely shorter than the noncancer COVID-19-positive patients. **Supplementary Table S5** shows the recently published estimated mean incubation period and serial interval for COVID-19-positive noncancer patients. A heatmap presentation of disease progression for 120 noncancer and 64 cancer cases shows that COVID-19-related symptom was 3.48 ± 2.17 days (mean ± SD) after first possible exposure to virus, and most cases were confirmed in 2.43 ± 1.47 days (mean ± SD) after the symptom onset (**Figures 2B, C**). The mean length of hospital stay for noncancer cases was 13.6 ± 7.27 (mean ± SD), and 16.11 ± 8.69 (mean ± SD) before the individuals were either recovered, discharged, or deceased (**Figures 2B, C**). The mean serial interval of the fitted normal distribution is 5.09 days (95% CI: 4.87–6.07) days for noncancer patients, while it was 3.69 days (95% CI: 3.02–4.39) for cancer cases (**Figures 3A–C** and **Supplementary Tables S3, S4, S6**; **Supplementary Figure S2**).

### SARS-CoV-2 Viral Load Is Higher in Cancer Patients Than Noncancer Patients

We report SARS-CoV-2 viral load analysis from two commercial viral detection assay kits (details in “*Materials and Methods*” section). We analyzed the impact of SARS-CoV-2 viral load for all 64 cancer patients as well as 120 noncancer patients with confirmed COVID-19 diagnosis. All patients were tested using nasopharyngeal swabs. To investigate the impact of SARS-CoV-2 viral load, we obtained the Ct values for SARS-CoV-2-specific gene targets quantified using two separate assay which specifically target SARS-CoV-2. We first compared the results with that of Roche’s cobas SARS-CoV-2 ORF-1ab and E gene. Results show that MOLgen-SARS-CoV-2 and Sansure Biotech SARS-CoV-2 N gene is highly correlated with that of the Roche cobas ORF-1ab and E genes, and no significant Ct value variations were found among the two detection kits (**Supplementary Figure S3**).

**Figure 4:**
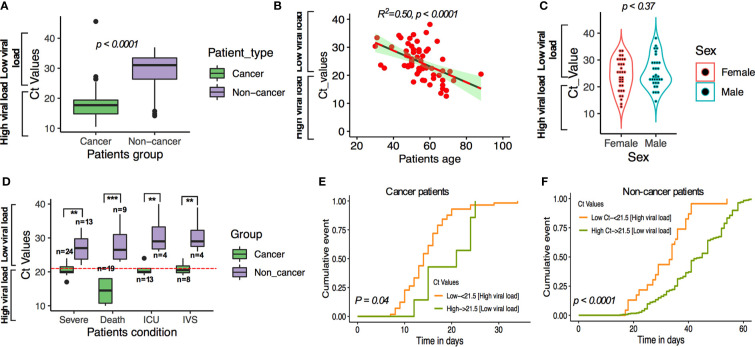
**(A)** SARS-CoV-2 Ct (cycle threshold) values in cancer and non-cancer patients derived from nasopharyngeal swab test. **(B)** Linear regression of age and viral load. **(C)** t-test comparing the viral load among male and female cases. **(D–F)** SARS-CoV-2-viral load and patients’ severe condition in patients with and without cancer. **P ≤ 0.01; ***P ≤ 0.001.

We transformed the Ct value into quantitative assessment of viral load. For this, Ct values were divided into three quartiles Q1, Q2, and Q3. Q1 represents the high viral load (Ct values less than 21.5; and viral load 5.30–5.778 log_10_ RNA copies/mL), Q2 medium viral load (Ct value between 21.5 and 27.3; 3.70 and 5.30 log_10_ RNA copies/mL) and low viral load (Ct values over 27.3; 2.67–3.69 log_10_ RNARoche cobascopies/ml). The median Ct values for the SARS-CoV-2-specific gene target for cancer (MOLgen detection assay) was 21.5 (IQR: 17.3–29.57) compared with a median Ct value of over 23.7 (IQR = 22.24–32.4) in noncancer patients (**Figure 4A**). Patients’ age has shown a significant positive correlation with high viral load (**Figure 4B**, *p* < 0.0001; *R* ^2^ = 0.50), but there was no significant difference in viral load between male and female (**Figure 4C**, *p* = 0.32).

Using this cutoff, compared with COVID-19 patients without cancer and low viral load, cancer patients and high viral load had higher observed severe patient’s condition (OR:1.42; 95% CI: 1.17–3.003; *p* < 0.001), higher death (OR: 1.62; 95% CI: 1.27–3.25; *p* = 0.0016), ICU admission (OR: 1.25; 95% CI: 1.07–2.81, *p* = 0.0029), and higher chances of requiring IVS (OR: 1.10; 95% CI: 0.91–2.38; *p* = 0.098; **Figure 4D**; **Supplementary Table S7**). We then analyzed the possible occurrence of severity, death, ICU admission and IVS requirement in cancer and noncancer patients’ group by cumulative survival analysis. We found that the severe condition occurred earlier in cancer patients where SARS-CoV-2 viral load is higher than the low SARS-CoV-2 viral load in noncancer patients (**Figures 4E, F**). These results are consistent with results obtained for multivariate regression results after adjusting for patients underlying medical conditions (**Supplementary Tables S1A–C**).

### High Viral Load Is Associated With Severe Condition and High Mortality in Certain Type of Cancer Patients

**Figure 5:**
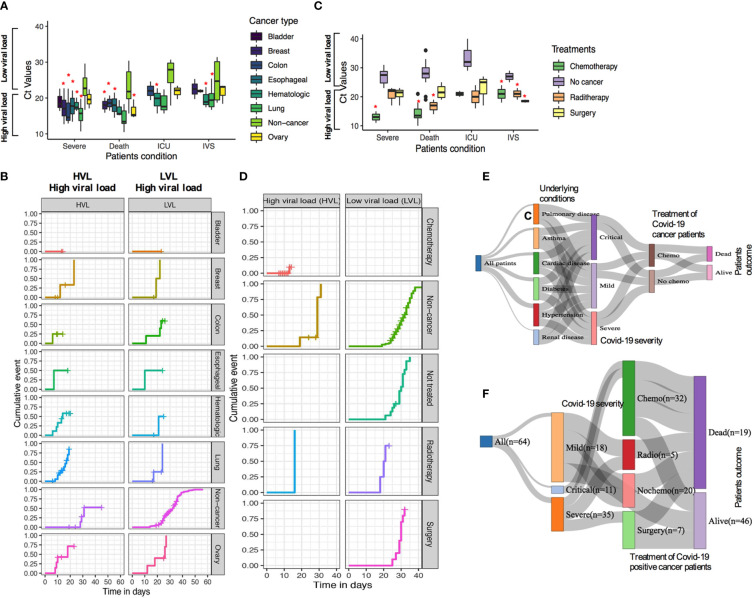
**(A, B)** SARS-CoV-2 Ct (cycle threshold) values in different type of cancer patients derived from nasopharyngeal swab test **(C, D)**. Relationship of chemotherapy use of confirmed Covid-19 cases and the mortality and severity of disease outcome. **(E, F)** Relationship of patients underlying condition, disease severity, and chemotherapy treatment and patient’s outcome. *P ≤ 0.05.

Next, we sought to compare SARS-CoV-2 viral load and patient’s severity and mortality in several cancer type and noncancer cohort. The mortality rate was 41.38% for high viral load; 23.81% in medium viral load; and 14.29% in low viral load cancer patient with COVID-19 infection, while the mortality was lower in noncancer COVID-19-infected patients (22.22% for high viral load, 5.13% in medium viral load, and 1.85% in low viral load patients; **Table 2**). We then compared viral load analysis between the different types of cancers and noncancer patients using similar cutoff criteria as those above. As shown in **Figures 5A, B**, lung, colon, hematologic, esophageal, and breast cancer presented high SARS-CoV-2 viral load with higher risk of severe patients’ conditions, have relatively high death rate, ICU admission, and comparatively higher chance of requiring IVS in the shortest possible time.

**Table 2:** Mortality rate of cancer and noncancer patients based on SARS-CoV-2 viral load.

Noncancer patients who are COVID-19 positive	
***Viral load***	***% Mortality***
Low (*n* = 54)	(*n* = 1; 1.85%)
Medium (*n* = 39)	(*n* = 2; 5.13%)
High (*n* = 27)	(*n* = 6; 22.22%)
***Cancer patients who are COVID-19 positive***	
Low (*n* = 14)	(*n* = 2; 14.29%)
Medium (*n* = 21)	(*n* = 5; 23.81%)
High (*n* = 29)	(*n* = 12; 41.38%)

Among the 64 COVID-19-positive cancer cases, patients have received different therapeutic regimens, such as, chemotherapy, surgery, or radiotherapy treatments. In total, 33 (33/64; 51.6%) had chemotherapy, 5 (5/64; 7.8%) had radiotherapy, 10 (10/64; 15.6%) had surgery, and 16 (16/64; 25.0%) had surgery before these patients were tested positive for COVID-19 (**Supplementary Table S8**). Patients who received chemotherapy tended to show a high rate of developing severe conditions in patients with high SARS-CoV-2 viral load compared with low viral load (**Figures 5C, D**). However, radiotherapy and surgery had medium to no effects in developing severe conditions with lower low viral load. When mortality/death was assessed, chemotherapy-, surgery-, and radiotherapy-treated patients having high viral load lead to a higher rate of death, higher chances of ICU admission, and higher use of IVS (**Figures 5C, D**). Additionally, the relationship between patients who received chemotherapy and underlying conditions and disease severity as well as disease outcome for COVID-19-positive cases are shown in **Figures 5E, F**.

## Discussion

Our study demonstrated that the differences in SARS-CoV-2 viral load in COVID-19-positive cancer and noncancer patients may play a role in the prediction of mortality and the extent of disease severity. SARS-CoV-2 viral load is significantly higher in cancer patients with increasing disease severity and mortality compared with noncancer patients. Several observations have emerged from our study. First, cancer patients with active COVID-19 infection showed shorter incubation period and serial interval time when compared with noncancer patients. Secondly, patients with cancer infected with COVID-19 and high viral load are more likely to experience severe and deleterious outcomes compared with patients with noncancer and low viral load. Thirdly, lung and breast cancer patients with high viral load demonstrated higher gravity of severe events, i.e., death, ICU support, and invasive ventilation requirement compared with noncancer and low viral load individuals. Lastly, cancer patients who are under active anticancer treatment or have been previously treated with anticancer agents, particularly chemotherapeutic treatment, showed higher death rate and higher chances of experiencing critical symptoms due to high SARS-CoV-2 viral load. These results may be useful for the consideration of prognostic tools to monitor and stratify the patients for delivering relevant treatment.

Since the emergence of COVID-19, the virus has rapidly spread all over the world and many countries are grappling in their search of epidemiological characteristics and control of the transmission of the virus, including its assessment of overall outcome and its impact in the society. An important observation of the COVID-19 pandemic is that elderly males reported severe disease and higher mortality than females (16–20). Moreover, age, smoking status, and comorbidities, such as, hypertension and cardiovascular disease are the risk factors for severe disease and mortality among patients with cancer and noncancer COVID-19 patients (8, 21). Since the start of the global pandemic of COVID-19, patients with cancer in most countries became the center point of concern due to their higher vulnerability, increased risk of contracting COVID-19, severe outcomes, and comparatively higher rate of requiring intensive care and increased risk of death (7, 22). Given the similar epidemiological features between cancer and noncancer COVID-19 patients, it is highly likely that cancer patients are endowed with some additional features different from noncancer individuals. Considerably wide gaps remain in our understanding of SARS-CoV-2 pathogenesis, including whether levels of viral load and disease severity and deaths in patients with cancer. To ascertain these additional attributes, this study was conducted to find out the possible effects of SARS-CoV-2 viral load in cancer patients and possible outcomes. Our study evaluating the patient’s severe condition and death not only confirm the association of higher-level viral load with these factors, but these factors have an additive effect which may contribute to the increased risk of mortality and severe condition in patients with multiple risk factors.

Perhaps the most important observation in our study is that COVID-19 patients with cancer and high viral load had significantly more severe outcomes, increased number of deaths, higher rates and longer stay in the hospitals and ICU, and invasive ventilation supports compared with low viral load and noncancer patients, confirming a fraction of previously published reports (2, 7). The outcome of COVID-19-positive patients without cancer was characterized by relatively lower death rate (2%–3%) (2), in contrast to the rate of death in COVID-19-positive cancer patients that was exceeded considerably (threefold), suggesting the vulnerability of cancer patients during the pandemic (2). In our study, we did observe a substantial impact of high SARS-CoV-2 viral load on the cancer patient’s death rate and disease severity, which may suggest that distinct factors modulating COVID-19 susceptibility and related outcome to the cancer patients (2, 3, 23).

The outcome of COVID-19-positive noncancer and cancer patients was also characterized by patients’ underlying conditions. Patients experiencing one or more underlying condition are likely to exhibit a more severe form of COVID-19 symptoms. In a large cohort study (*n* = 22,753), it was reported that, major underlying conditions were cardiovascular disease (8.9%), hypertension (27.4%), diabetes (17.4%), and cancer (3.5%) (21). This suggests that presence of any of these underlying conditions and high viral load may severely affect the clinical outcome of patients with COVID-19 (24). Our findings are consistent with those presented in the previous studies suggesting the potentially additive adverse effects of one or more underlying conditions of COVID-19-positive cancer patients with high SARS-CoV-2 viral load.

We described a cohort of cancer patients with confirmed COVID-19 positivity and explored the association of viral load and outcome of cytotoxic chemotherapy. Our cohort includes breast (25.0%), lung (14.06%), head and neck (9.38%), bladder (9.38%), colon (10.94%), and ovarian (7.81%) cancers. We identified that all cancer patients with high viral load experienced the severe symptom of COVID-19 with a life-threatening possibility and death. A substantial proportion of death was observed in severe and critical cases. The mortality rate is higher than the low viral load noncancer patient’s cohort which may characterize the severity of the symptoms of cancer patients with high viral load. The rate of hospitalization and those seeking ICU and invasive ventilation support was much higher than those of noncancer patients, which was approximately 29.41% vs. 1.67% and 17.65% vs. 0.0%, respectively. This finding thus suggests that having a detection of COVID-19 on cancer patients with high viral load substantially increases the burden of hospitalization and utilization of more intensive care supports. From our analysis, it was observed that patients with lung cancer had a higher death rate followed by breast cancer among all patients with cancer. The average time to death was shorter and had a rapidly deteriorating clinical condition once tested positive for COVID-19 who upon admission to a hospital detected high SARS-CoV-2 viral load. This adverse clinical condition may be due to high viral load for most cancer patients. We speculate that compromised immune systems due to cytotoxic chemotherapy may play a role in impeding the immunologic functions and thereby deteriorating clinical conditions for cancer patient’s death. Although our small sample size limits us to draw a definitive conclusion about our observation, it could be used as a reference point along with other larger cohort studies (2, 3, 23, 25, 26). A recent report showed that patients with hematologic and metastatic lung cancer had the highest frequency of severe events (2). Our results are consistent with this report at least for lung cancer, since cancer cohort is smaller in hematologic malignancy cases thus limiting our understanding of severe events on hematologic cancer.

In our cohort, patients with cancer mainly received two major cancer-related treatments and therapies including chemotherapy and radiotherapy. Outcome of cancer patient’s treatment tested positive for COVID-19 was characterized by higher rate of death and severity. Patients receiving chemotherapy within 4–6 months before confirmed COVID-19 infection had the highest rate of mortality and severity of illness, which strikingly contrasts to recently published results (23). Several recent studies reported that hematological malignancies and patients receiving immunotherapies had the highest death rate and severe clinical phenotypes (2, 3, 23). Our study lacks patients who receive targeted therapy and immunotherapy, and we cannot conclude that only cytotoxic chemotherapy is solely responsible for higher mortality and severe patients’ characteristics, however, likely increase the chances of severity and mortality due to immunocompromised conditions postchemotherapy. A larger cohort with a variety of treatment strategies may precisely answer our observations.

Finally, we analyzed the serial interval and incubation period differences between noncancer and cancer cohorts, which are key parameters for transmission modeling and assists governments and policy makers to respond to the pandemic. These two parameters influence the disease incidence and prevalence of transmission (13, 27). In the noncancer and cancer cohorts, we estimated the serial interval and obtained a shorter serial interval (3.9 days) for cancer patients’ cohort, while the noncancer patients’ serial interval was 5.09 days (13). Furthermore, the estimated serial interval is shorter than the incubation period in cancer cohorts suggesting a presymptomatic transmission for cancer patients.

As usual, our study has several limitations. Although our study cohort for cancer is comparatively smaller than recently published studies and only symptomatic cases were included who seek help from healthcare providers, larger cohort as well as asymptomatic cases, and hidden cases who did not report for assistance could further extend our understandings of actual characteristics of cancer patients’ treatment and COVID-19 infection. Furthermore, due to the government pandemic restrictions, it was not possible to collect all the available patient’s data and records from the healthcare providers which potentially impeded our analysis. With some larger number of patient’s analysis, we would be able to pinpoint more unanswered questions. Despite all these limitations, our study is unique when compared with the larger studies, a representative of small numbers of cancer patients among hundreds of unidentified cases. Future work with diverse cancer type and larger patients’ cohort and long-term follow-ups will define the specific risk of COVID-19 on outcomes in much greater gravity in patients with cancer.

## Data Availability Statement

The raw data supporting the conclusions of this article will be made available by the authors, without undue reservation.

## Ethics Statement

This study was approved by the central ethics committee of Bangladesh Medical Research Council/Park View Hospital (study # 2021-2023/62(1-20) and King Faisal Specialist Hospital and Research Centre (RAC # 2200031)- a waiver of informed consent from patients was also approved. All research was performed in accordance with the relevant ethical guidelines and regulations. Written informed consent for participation was not required for this study in accordance with the national legislation and the institutional requirements.

## Author Contributions

SI contributed to the conception and design of the study. SI and MA-M contributed in data analysis. SI, MA-M, and AN contributed to writing the manuscript. SI, AN, KA, MA, JA, RK, AZ, and MK have contributed to literature search, data collection, data interpretation, and clinical data inconsistencies. All authors contributed to the article and approved the submitted version.

## Funding

This study was supported by King Faisal Specialist Hospital and Research Centre internal grant (Grant no. RAC-2200031). The funding agency had no roles in the study design, data collection, data interpretation, and writing of the manuscript.

## Conflict of Interest

The authors declare that the research was conducted in the absence of any commercial or financial relationships that could be construed as a potential conflict of interest.

## Publisher’s Note

All claims expressed in this article are solely those of the authors and do not necessarily represent those of their affiliated organizations, or those of the publisher, the editors and the reviewers. Any product that may be evaluated in this article, or claim that may be made by its manufacturer, is not guaranteed or endorsed by the publisher.

## Supplementary Material

Supplementary Figure 1 **(A)** Case fatality ratio, **(B)** ICU admission, **(C)** number of severe patients, and **(D)** number of cases with invasive mechanical ventilation support among non-cancer and cancer patients at different age distributions. F. Forest plot showing the effects of COVID-19 positive patients underlying conditions and mortality. Whiskers indicated 95% CI.

Supplementary Figure 2 **(A, B)** Kaplan-Meier COVID-19 incubation period for all non-cancer and cancer cases.

Supplementary Figure 3 Correlation of MOLgen SARS-CoV-2 N1and Sansure SARS-CoV-2 N2 viral load assay with Cobas Roche SARS-CoV-2 ORF-1aba and E gene Ct values. P values derived from regression R2.
