# Supplementary figures and images for: SARS-CoV-2 Viral Load Is Correlated With the Disease Severity and Mortality in Patients With Cancer
Source: Front Oncol. 2021 Aug 18;11:715794. doi: 10.3389/fonc.2021.715794 (PMC8416515; doi:10.3389/fonc.2021.715794)

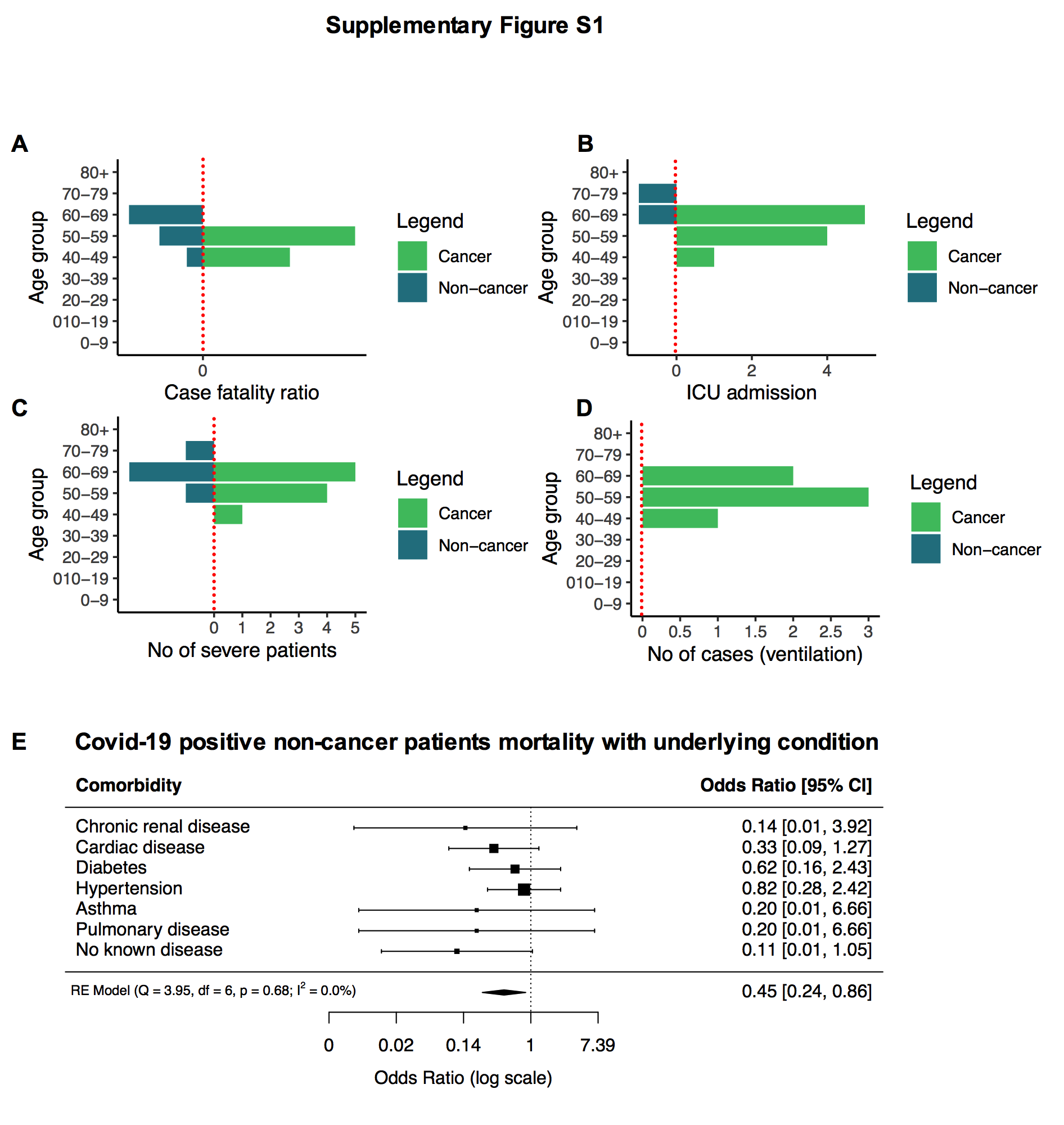

Supplement: Supplementary Figure 1 — (A) Case fatality ratio, (B) ICU admission, (C) number of severe patients, and (D) number of cases with invasive mechanical ventilation support among non-cancer and cancer patients at different age distributions. F. Forest plot showing the effects of COVID-19 positive patients underlying conditions and mortality. Whiskers indicated 95% CI. [file Image_1.tiff]

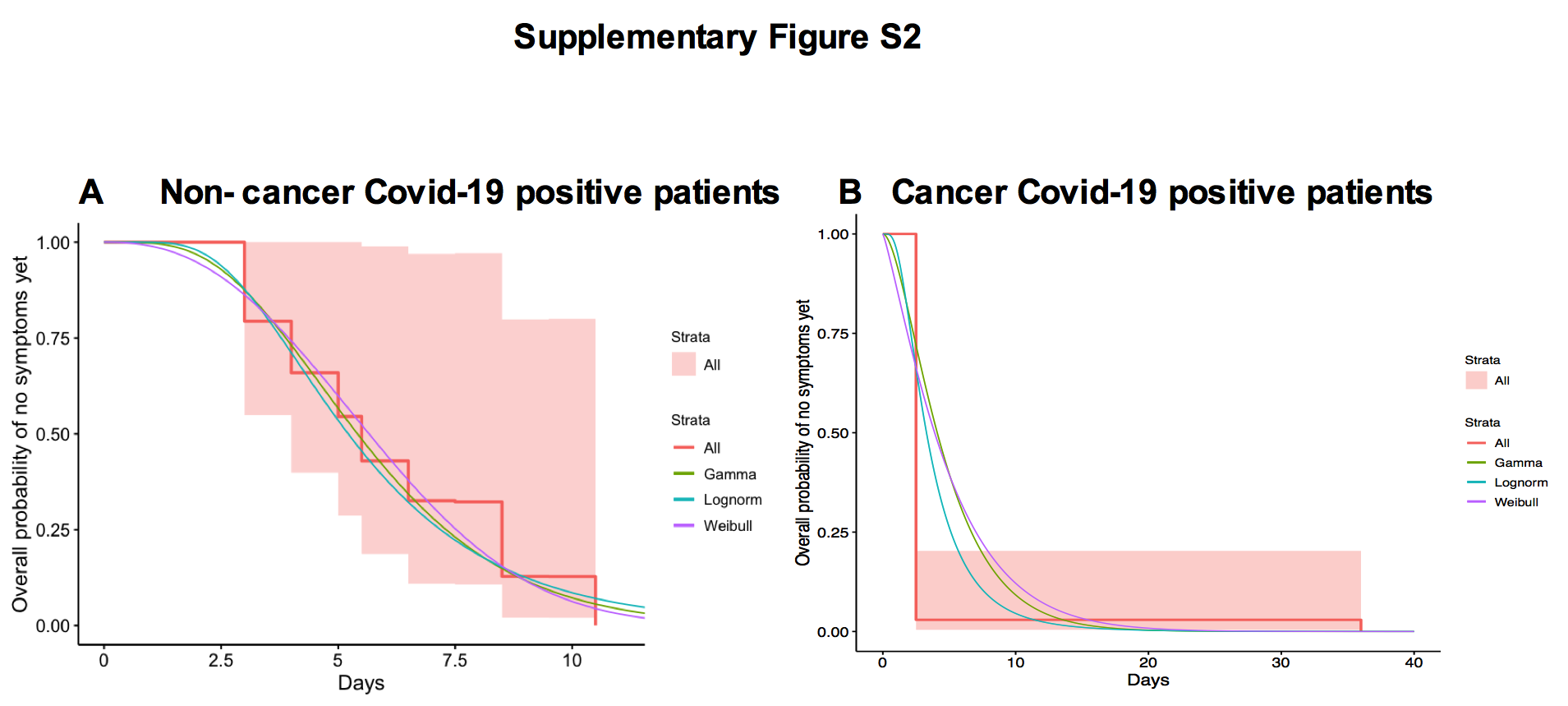

Supplement: Supplementary Figure 2 — (A, B) Kaplan-Meier COVID-19 incubation period for all non-cancer and cancer cases. [file Image_2.tiff]

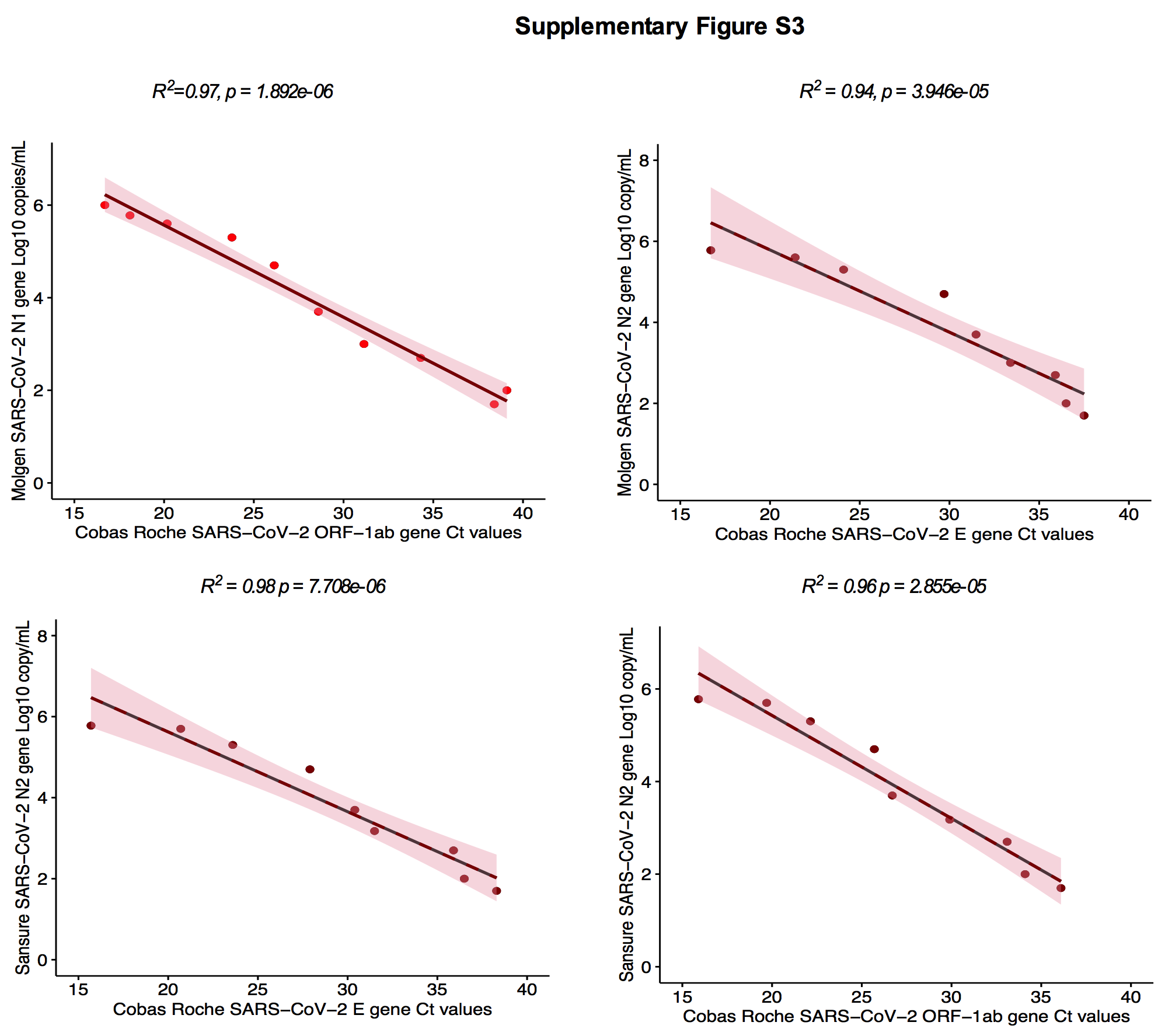

Supplement: Supplementary Figure 3 — Correlation of MOLgen SARS-CoV-2 N1and Sansure SARS-CoV-2 N2 viral load assay with Cobas Roche SARS-CoV-2 ORF-1aba and E gene Ct values. P values derived from regression R2. [file Image_3.tiff]
