# Supplementary material for: SARS-CoV-2 Viral Load Is Correlated With the Disease Severity and Mortality in Patients With Cancer
Source: Front Oncol. 2021 Aug 18;11:715794. doi: 10.3389/fonc.2021.715794 (PMC8416515; doi:10.3389/fonc.2021.715794)
Supplement: Supplementary file 4 [file DataSheet_1.zip › Supplementary Table 1.DOCX]

Supplementary table S1a: Multivariate-regression analysis of Covid-19 patients.

| ***Death*** | ***Underlying conditions*** | ***Odds ratio (95% CI)*** | ***p-values*** |
| --- | --- | --- | --- |
|  | ***Cancer*** | ***2.59 (1.47-5.26)*** | ***0.001*** |
|  | Chronic renal disease | 0.68 (0.42-1.04) | 0.46 |
|  | Cardiac disease | 1.53 (0.33-2.95) | 0.03 |
|  | Diabetes | 1.37 (0.71-2.32) | 0.02 |
|  | Hypertension | 1.21 (0.84-2.91) | 0.01 |
|  | Asthma | 0.43 (0.21-1.40) | 1.00 |
|  | Pulmonary disease | 1.13 (0.43-2.99) | 0.08 |

| ***ICU*** | ***Underlying conditions*** | ***Odds ratio (95% CI)*** | ***p-values*** |
| --- | --- | --- | --- |
|  | ***Cancer*** | ***4.22 (3.17-6.26)*** | ***0.001*** |
|  | Chronic renal disease | 1.08 (0.62-1.04) | 0.46 |
|  | Cardiac disease | 2.13 (1.33-4.95) | 0.03 |
|  | Diabetes | 1.17 (0.71-2.32) | 0.2 |
|  | Hypertension | 1.09 (0.84-2.91) | 0.13 |
|  | Asthma | 0.59 (0.21-1.40) | 1.00 |
|  | Pulmonary disease | 0.43 (0.13-1.99) | 0.8 |

| ***Severity*** | ***Underlying conditions*** | ***Odds ratio (95% CI)*** | ***p-values*** |
| --- | --- | --- | --- |
|  | ***Cancer*** | ***2.21 (1.77-4.61)*** | ***0.01*** |
|  | Chronic renal disease | 0.79 (0.42-2.41) | 0.53 |
|  | Cardiac disease | 1.33 (0.78-3.05) | 0.02 |
|  | Diabetes | 1.87 (1.21-3.28) | 0.02 |
|  | Hypertension | 2.11 (1.14-4.74) | 0.0013 |
|  | Asthma | 0.59(0.31-1.64) | 1.00 |
|  | Pulmonary disease | 0.83 (0.47-1.89) | 0.06 |

Supplementary table S1b: Multivariate-regression analysis of Covid-19 patients.

Supplementary table S1c: Multivariate-regression analysis of Covid-19 patients.
