# Supplementary material for: SARS-CoV-2 Viral Load Is Correlated With the Disease Severity and Mortality in Patients With Cancer
Source: Front Oncol. 2021 Aug 18;11:715794. doi: 10.3389/fonc.2021.715794 (PMC8416515; doi:10.3389/fonc.2021.715794)
Supplement: Supplementary file 4 [file DataSheet_1.zip › Supplementary Table 2.DOCX]

| Supplementary Table S2: Covid-19 positive cancer patients and severity, no of deaths, average time spent in hospital and ICU and invasive ventilation supports of each type of cancer | | | | | | |
| --- | --- | --- | --- | --- | --- | --- |
| ***Types of cancer*** | ***Numbers***  ***(%)*** | ***N***  ***(% deaths)*** | ***Average time between Covid-19 disease onset to death (days)*** | ***Average time spent in hospital*** | ***Average time spent in ICU (days)*** | ***Invasive ventilation support (days)*** |
| ***All cancers*** | ***64/100*** | ***19/29.69*** | ***17.6*** | ***23.8*** | ***7.2*** | ***6.0*** |
| ***NSCLC* (non- metastatic)*** | 4/11.76 | 3/15.79 | 11.0 | 8.0 | 9.0 | 7.0 |
| ***NSCLC (metastatic)*** | 5/14.71 | 4/21.05 | 10.0 | 8.0 | 8.0 | 5.0 |
| ***Hematologic cancer*** | 3/4.68 | 3/15.79 | 10.0 | 10 | NA | NA |
| ***Breast cancer (non-metastatic)*** | 5/7.81 | 0 | 16.0 | 13.0 | 6.0 | 6.0 |
| ***Breast cancer (metastatic)*** | 11/17.19 | 2/10.52 | NA | 13.0 | 9.0 | 7.0 |
| ***Ovarian cancer (metastatic)*** | 4/6.75 | 3/15.79 | 12.0 | 12.0 | 3.0 | 7.0 |
| ***Ovarian cancer (non-metastatic)*** | 1/1.56 | 0 | NA | 11.0 | 5.0 | NA |
| ***Bladder cancer*** | 6/9.38 | 0 | NA | 26.0 | NA | NA |
| ***Colon cancer (non-metastatic)*** | 7/10.94 | 2/10.53 | 14.0 | 5.0 | NA | NA |
| ***HNSCC** (non-metastatic)*** | 5/14.71 | 0 | NA | 12.0 | NA | NA |
| ***Esophageal*** | 4/6.25 | 2/10.52 | NA | NA | NA | NA |
| ***Rectal*** | 5/7.81 | 0 | NA | NA | NA | NA |
| ***Pancreas*** | 3/4.69 | 0 | NA | NA | NA | NA |
| ****NSCLC: Non-small cell lung carcinoma; **HNSCC: Head and neck squamous cell carcinoma*** | | | | | | |
