# Supplementary material for: SARS-CoV-2 Viral Load Is Correlated With the Disease Severity and Mortality in Patients With Cancer
Source: Front Oncol. 2021 Aug 18;11:715794. doi: 10.3389/fonc.2021.715794 (PMC8416515; doi:10.3389/fonc.2021.715794)
Supplement: Supplementary file 4 [file DataSheet_1.zip › Supplementary Table 3.DOCX]

**Supplementary table S3**. Mean incubation period and serial interval for Covid-19 positive non-cancer and cancer patients. Incubation period was based on gamma estimates. 95% CI are provided in

brackets.

| ***Source*** | ***Incubation time (days)*** | ***Serial interval time (days)*** | ***Mean time difference (days)*** |
| --- | --- | --- | --- |
| Non-cancer (all) | 5.81 (5.17-6.50) | 5.09 (4.87-6.07) | 0.72 |
| Cancer (all) | 4.99 (3.60-6.71) | 3.69 (3.02-4.39) | 1.30 |
