# Supplementary material for: SARS-CoV-2 Viral Load Is Correlated With the Disease Severity and Mortality in Patients With Cancer
Source: Front Oncol. 2021 Aug 18;11:715794. doi: 10.3389/fonc.2021.715794 (PMC8416515; doi:10.3389/fonc.2021.715794)
Supplement: Supplementary file 4 [file DataSheet_1.zip › Supplementary Table 4.DOCX]

**Supplementary table S4.** Estimation of incubation period using gamma, Weibull and log normal distributions for Covid-19 positive non-cancer and cancer patients. 95% CI are provided in the brackets for the parameters of shape and scale (log mean and sd for log normal are shown in the brackets)

| ***Distributions*** | ***Median*** | ***Parameter 1 (Shape)*** | ***Parameter 2 (Scale)*** |
| --- | --- | --- | --- |
| ***Gamma*** | | | |
| Non-cancer (all) | 5.41 | 4.84 (3.41-5.93) | 1.20 (0.81-1.34) |
| Cancer (all) | 4.10 | 1.89 (1.32-2.42) | 2.61(1.47-3.79) |
| ***Weibull*** | | | |
| Non-cancer | 5.66 | 2.43 (1.99-2.98) | 6.58 (5.89-7.35) |
| Cancer | 5.14 | 1.18 (0.92-1.52) | 5.32 (3.67-7.71) |
| ***Log normal*** |  | ***Log mean*** | ***Standard deviation*** |
| Non-cancer (all) | 5.22 | 1.65 (1.54-1.85) | 0.476 (0.397-0.571) |
| Cancer (all) | 4.20 | 1.19 (0.85-1.44) | 0.658 (0.493-0.877) |
