# Supplementary material for: SARS-CoV-2 Viral Load Is Correlated With the Disease Severity and Mortality in Patients With Cancer
Source: Front Oncol. 2021 Aug 18;11:715794. doi: 10.3389/fonc.2021.715794 (PMC8416515; doi:10.3389/fonc.2021.715794)
Supplement: Supplementary file 4 [file DataSheet_1.zip › Supplementary Table 5.DOCX]

Supplementary Table S5: Estimated mean incubation period and mean serial interval of Covid-19 in non-cancer cases from recently published studies.

| **Data source** | **Number of cases reported** | **Mean incubation period (days)** | **Mean serial interval (days)** | **References** |
| --- | --- | --- | --- | --- |
| Singapore | 93 | 4.91 (4.35-5.69) | 4.17 (2.44-5.89) | Tindale et al 2020 |
| Tianjin | 135 | 7.54 (6.76-8.56) | 4.31 (2.91-5.52) | Tindale et al. 2020 |
| Infector-infectee pairs | 28 pairs | na | 4.0 (3.1-4.9) | Nishiura et al 2020 |
| Outside Wuhan cases | 158 | 5.6 (5.0-6.3) | na | Linton et al. 2020 |
| Hong Kong Transmission chains | 21 chains | na | 4.4 (2.9-6.7) | Zhao et al. 2020 |
| Wuhan travelers | 88 | 6.4 (5.6-7.7) | na | Backer et al. 2020 |
