# Supplementary material for: SARS-CoV-2 Viral Load Is Correlated With the Disease Severity and Mortality in Patients With Cancer
Source: Front Oncol. 2021 Aug 18;11:715794. doi: 10.3389/fonc.2021.715794 (PMC8416515; doi:10.3389/fonc.2021.715794)
Supplement: Supplementary file 4 [file DataSheet_1.zip › Supplementary Table 6.DOCX]

| **Non-cancer** | | | |
| --- | --- | --- | --- |
| ***Distributions*** | ***Mean/Shape*** | ***SD/Rate/Scale*** | ***AIC*** |
| Log normal | 1.52 (1.44-1.60) | 0.42 (0.38-0.48) | 514.41 |
| Gamma | 5.24 (4.21-6.87) | 1.03 (0.82-1.36) | 525.80 |
| Weibull | 2.15 (1.88-2.48) | 5.76 (5.26-6.29) | 548.22 |
| **Cancer** | | | |
| Log normal | 1.25 (1.13-1.38) | 0.35 (0.26-0.44) | 99.21 |
| Gamma | 9.51 (6.14-18.66) | 2.73 (1.66-5.11) | 94.61 |
| Weibull | 4.13 (3.16-5.68) | 4.05 (3.70-4.42) | 89.08 |

**Supplementary table S6.** Serial interval estimation and model comparison of Covid-19 positive non-cancer and cancer patients
