# Supplementary material for: SARS-CoV-2 Viral Load Is Correlated With the Disease Severity and Mortality in Patients With Cancer
Source: Front Oncol. 2021 Aug 18;11:715794. doi: 10.3389/fonc.2021.715794 (PMC8416515; doi:10.3389/fonc.2021.715794)
Supplement: Supplementary file 4 [file DataSheet_1.zip › Supplementary Table 7.DOCX]

| Supplementary table S7. Comparison of different factors associated with High SARS-CoV-2 viral load. | | | | |
| --- | --- | --- | --- | --- |
|  | Odds ratio; [95% CI]; p-value | | | |
| Variables | Severity | Death | ICU | IMV |
| Cancer vs non-cancer | 1.42 [1.17-3.003]  P < 0.001 | 1.60 [1.27-3.25]  p = 0.0016 | 1.25 [1.07-2.81]  p = 0.0029 | 1.10 [0.91-2.38]  p = 0.098 |
