# Supplementary material for: SARS-CoV-2 Viral Load Is Correlated With the Disease Severity and Mortality in Patients With Cancer
Source: Front Oncol. 2021 Aug 18;11:715794. doi: 10.3389/fonc.2021.715794 (PMC8416515; doi:10.3389/fonc.2021.715794)
Supplement: Supplementary file 4 [file DataSheet_1.zip › Supplementary Table 8.DOCX]

| Supplementary table S8: Clinical characteristics and outcome of Covid-19 positive cancer patients. | | | |  |
| --- | --- | --- | --- | --- |
| ***Patient no.*** | ***Sex*** | ***Cancer type*** | ***Phase of cancer treatment*** | ***Survival status***  ***Deceased – 19 cases***  ***Alive – 45 cases*** |
| 1, 4 | M, M | NSCLC | Thoracic radiotherapy (27 Gy in 9 fr) | # 3, 2, 6, 9, 5, 7, 8-deceased |
| 3 | M |  | Chemotherapy regime: Cisplatin and radiotherapy |  |
| 2, 5, 6, 9, | M, M, M, M |  | 2-cycle of adjuvant chemotherapy(cisplatin/docetaxel) before surgery |  |
| 7, 8 | F, M |  | Follow-up for 1-year post-chemotherapy |  |
| 10,14,12, 16  11,15,13  18, 17, 1917,19, | F,F,F,F  F,F,F  F, F, F | Breast cancer | Adjuvant chemotherapy | # 13, 19 deceased |
|  |  |  | Adjuvant chemotherapy |  |
|  |  |  | Adjuvant chemotherapy |  |
|  |  |  | Adjuvant chemotherapy |  |
| 20, 21, 22, 23, 24 | F,F,F,F,F |  | Surgery |  |
| 25 | F |  | Newly diagnosed (waiting for treatment to be commenced). |  |
| 26, 27, 28, 29, 30 | F, F, F, F | Ovary | 3-cycle of chemotherapy (cisplatin/taxane) | # 26, 28, 30 deceased |
| 31,32,33 | M,M,M, | Bladder | Radiotherapy |  |
| 34,35,36 | M, M, M | Bladder | Surgery |  |
| 37,39,40 |  | Esophageal | Best supportive care | # 40 deceased |
| 38 |  | Esophageal | Chemotherapy | # 38 deceased |
| 41, 43 |  | Pancreas | Surgery |  |
| 42 |  | Pancreas | Chemotherapy |  |
| 44,45,46,47,48 |  | Rectal | Not treated yet |  |
| 49,50,51 | M, M, F | Hematologic | Chemotherapy | # 49, 50, 51 deceased |
| 52 | M | Colon | Best supportive care | # 55,58 deceased |
| 53,54,55,58 | M,M,M |  | Chemotherapy |  |
| 56,57 | M,M | Colon | No treatment started yet |  |
| 59,60,61,62 | M,M,F | HNSCC | Best supportive care |  |
| 63,64 | M,M |  | Chemotherapy regime: Cisplatin and radiotherapy |  |
| ***M-Male, F-Female; NSCLC-Non-small cell lung carcinoma; HNSCC-head and neck squamous cell carcinoma*** | | | | |
